# Comparison of bayberry fermented wine aroma from different cultivars by GC‐MS combined with electronic nose analysis

**DOI:** 10.1002/fsn3.1343

**Published:** 2020-01-09

**Authors:** Yuxi Cao, Zufang Wu, Peifang Weng

**Affiliations:** ^1^ College of Food and Pharmaceutical Sciences Ningbo University Ningbo China; ^2^ Key Laboratory of Animal Protein Deep Processing Technology of Zhejiang Province Ningbo University Ningbo China

**Keywords:** bayberry cultivars, bayberry wine fermentation, electronic nose, GC‐MS, volatile compounds

## Abstract

Four bayberry cultivars (Biqi, Dongkui, Wandao, and Dingao) in eastern China were selected to produce the fermented bayberry wine. The volatile flavor compounds in different bayberry wine were compared by gas chromatography–mass spectrometry (GC‐MS) and electronic nose. The results showed that 46 volatile flavor compounds were found in bayberry wine, including 19 esters, 7 alcohols, 6 acids, 2 aldehydes, 2 ketones, 3 terpenes, and 7 others compounds. The most important contribution to the aroma of bayberry wine was esters and alcohols, respectively. Differentiation of four kinds of bayberry wine was conducted analysis by E‐nose. Sensory evaluation showed that Biqi bayberry wine was highly evaluated for its highest score in color, floral aroma, overall acceptability, and fruity aroma. Our results suggest that there were differences in the flavor characteristics of bayberry wine brewed from different varieties of bayberry. The results of this study will provide valuable information for bayberry wine makers to select raw materials.

## Graphical abstract

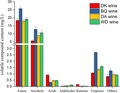

## 1 INTRODUCTION

Bayberry (*Myrica rubra*) originating from China is one of the most popular fruits on the market (Fang, Zhang, Sun, & Sun, 2006). It is cultivated in China for more than 2000 years (Chen, 1996). Bayberry is a favorable and profitable fruit with abundance in carbohydrate, organic acids, soluble sugars, minerals, vitamins, and phenolics (Cheng et al., 2016; Xu, Zhang, Fang, Sun, & Wang, 2014). Dongkui bayberry (DK), Biqi bayberry (BQ), Dingao bayberry (DA), and Wandao bayberry (WD) are the four main cultivars in Zhejiang province, China, accounting for more than 60% of the total yield of bayberry in China. Because bayberry was harvested during the hot and rainy season from June to July, it was susceptible to mechanical injury and microbiological decay (Fang et al., 2009; Yu, Lin, Zhan, He, & Zhu, 2013), which greatly affects the commercial value of the bayberry (Zhang et al., 2005). With the increase in yield of bayberry, the bayberry has been further processed into juice drinks (Shao & He, 2007), canned bayberry (Ya‐Mei et al., 2007), and dried bayberry (Cheng, Chen, Chen, et al., 2015) and bayberry wines in order to increase its consumption and extend the shelf life. Bayberry wine is produced by fermenting methods using bayberry as raw material has extremely high nutritional value and medicinal effect (Zhang, Li, & Fan, 2019).

Aroma is an important indicator that influences the intrinsic quality of bayberry fruit and its deep processed products. In addition, aroma was one of the most valuable attributes of wines that determines the sensory quality and value of wine (Mamede, 2005). The quality of aroma directly affects the flavor quality of bayberry wine and the consumers' acceptance and preference. Chinese bayberry cultivars grown in different locations have different flavors, which affect the flavor and quality of bayberry wine. Xu et al. (Xu et al., 2014) have studied the flavor changes during processing and storage of bayberry juice using the headspace solid‐phase microextraction (HS‐SPME) coupled with gas chromatography–mass spectrometry (GC‐MS). The results showed that the bayberry juice produced fermentation‐like flavors with an increase in alcohols (11.45%) and decreases in esters (14.91%) after 9 months of storage. Cheng et al. (Cheng, Chen, Li, et al., 2015) used HS‐SPME‐GC‐MS combined with principal component analysis to identify the volatile flavor components of bayberry during storage, indicating that different varieties of bayberry have different flavor characteristics. Kang et al. (Kang, Li, Xu, Jiang, & Tao, 2012) studied the aroma components of immature and mature bayberry fruit by HS‐SPME/GC–MS. The results showed that terpenoids (such as caryophyllene) were the most abundant, and alcohol, aldehydes, ketones, esters, and acids were less abundant. However, it has not yet been fully reported investigating the volatile flavor compounds of bayberry wines by GC‐MS coupled with electronic nose (E‐nose). GC‐MS studies have mainly focused on the measurement of certain volatile compounds, while the E‐nose is an instrument that uses chemical sensors to detect volatiles and then provides a holistic view of the volatile compounds of the sample through a powerful mathematical software analysis system that helps determine the odor. The detection data can be analyzed using principal component analysis (PCA), cluster analysis (CA), and linear discriminant analysis (LDA) (Wilson & Baietto, 2009). Owing to the advantages of rapid, accurate, and effective determination, well verification, and complement to each other, E‐nose combined with GC–MS had already been used in the analysis of Goji berries (Li, Yu, Xu, & Gao, 2017). In addition, E‐nose has been used to evaluate the quality of beverages (Banerjee, Tudu, Bandyopadhyay, & Bhattacharyya, 2019), including the identification of alcohol brands, the quality identification of distilled white spirits, and the identification of different types of red wines (García et al., 2006; Lozano, Arroyo, Santos, Cabellos, & Horrillo, 2008). However, little information has been reported in aroma analysis of different bayberry wines by using E‐nose combined with HS‐SPME/GC‐MS.

The fruit wine industry has a famous saying that “wines success for brewing, more important is raw materials,” which shows the importance of raw materials in fruit wine brewing (González‐Mas et al., 2009). There are differences in the characteristics and composition of different varieties of bayberry fruit, such as color, aroma, and taste (Cheng et al., 2016). The influence of bayberry cultivars on the volatile flavor compounds of bayberry wines has not been reported.

The choice of yeast is a key step in the production of fruit wine, which directly affects the flavor quality of the bayberry wine. The bayberry variety in China contains a large amount of malic acid to negatively affect the wine quality. *Issatchenkia orientalis* can degrade malic acid efficiently (Kim, Hong, & Park, 2008; Negi & Dey, 2013). In our previous study, two strains of *Saccharomyces cerevisiae* 131 (Sc 131) and *Issatchenkia orientalis* 166 (Io 166) were selected for mixed fermentation of fruit wines, which are the most suitable to produce flavor and alcohol components, respectively (Wenwen, Peifang, & Zufang, 2019). The present study aims to elucidate the flavor characteristics and key volatile components of four varieties of bayberry wines fermented by Io 166 and Sc 131. GC‐MS and E‐nose were used to analyze the volatile flavor components of four major main‐planted bayberry cultivars in eastern Zhejiang. At the same time, its components and sensory quality were also analyzed. This study results provided an important basis for the selection of bayberry cultivars and raw materials for fermenting wine.

## 2 MATERIALS AND METHODS

### 2.1 Sample preparation

Bayberry cultivars including Dongkui (DK), Biqi (BQ), Dingao (DA), and Wandao (WD) were used as raw materials for wine fermentation. These bayberries were purchased at a mature stage from their main production areas in Ningbo, Zhejiang Province, China, on the same day during June 2018. Bayberries were packaged in ice bags and transported to the laboratory as soon as possible, where they were preserved at 4°C no more than an hour. Then, four cultivars of bayberry juices were produced with a juicer extractor and filtered through gauze within 1 hr.

### 2.2 Chemicals and reagents

Ethanol Assay Kit K‐ETOH was purchased from Megazyme, Ireland; YPD medium, phenol, sodium hydroxide, potassium metabisulfite, 3,5‐dinitrosalicylic acid, sodium potassium tartrate, and sodium bisulfite are of analytical grade and they are purchased from Sinopharm Chemical Reagent Co., Ltd.

### 2.3 Winemaking process

The total soluble solid (TSS) content of bayberry juice was adjusted to 22.5 °Brix using sucrose. Then, potassium metabisulfite was added to make the concentration of sulfur dioxide at 40 ~ 100 mg/L. Each cultivar was well mixed before winemaking and separated into three replicates to avoid compositional variation (Liu, Li, Gao, Cheng, & Yuan, 2019). The mixture was pasteurized at 75°C for 15 min and cooled to 20°C and inoculated with Sc 131 at approximately 10^5^ cfu/ml and Io 166 at approximately 10^6^ cfu/ml. The main fermentation was conducted at 27°C for about 4 to 6 days until the total sugar content less than 8 g/L. Then, the fermented mash was placed at 18°C for 15 days for post‐fermentation. At the end of fermentation, the wine was clarified using 0.08 g/L of chitosan for 2 hr and racked for 1 day at 4°C. After centrifugation, 70 mg/L of potassium metabisulfite was added to the wines. Then, bayberry wines were bottled with equal headspace volume. Finally, they were labeled and stored at room temperature, respectively, for 3 months in the dark before analysis was carried out.

The concentration of each important composition was detected according to references including total sugars (Liu et al., 2013), total soluble solids (Koshita, Yamane, Yakushiji, Azuma, & Mitani, 2011), and total anthocyanin (Giusti & Wrolstad, 2001), as well as alcohol, titratable acid, and pH. Alcohol content was detected by Ethanol Assay Kit K‐ETOH. The analysis was conducted in quadruplicate for each parameter investigated.

### 2.4 GC‐MS analysis

According to the previously published method, the volatile compounds in the bayberry wine were extracted by headspace solid‐phase microextraction, and the method was slightly modified (Liu et al., 2018; Yu, Xie, Xie, Ai, & Tian, 2019). Bayberry wine samples (5 ml) were added into 20‐ml headspace glass vials (18 mm precision thread vial of preassembled cap and septa) with 1.5 g sodium chloride and 20 μL internal standard of 2‐octanol (10 mg/L). The purpose of adding sodium chloride is to promote the volatilization of volatile components. The vial was sealed with white silicone/blue PTFE septa and equilibrated in a constant temperature water bath at 40°C for 20 min. Then, it was desorbed into the GC inlet with the automatic autosampler within 7 min at 210°C. The 50/30 μm DVB/CAR/PDMS SPME fiber was inserted into the headspace of the vial to extract the volatile compounds in the bayberry wine at 40°C for 20 min. GC‐MS (Model 7890B‐7000C, Agilent Technologies) equipped with a nonpolar column (J&W Scientific DB‐5; 30 mm, ID 0.25 mm, film thickness 0.25 μm) was used to analyze the volatile compounds of bayberry wines. Helium was used as the carrier gas with the flow rate at 1.2 ml/min under a splitless GC inlet mode. The program of oven temperature was as follows: initial temperature 50°C for 3 min, rising to 200°C at the rate of 5°C/min and held at 200°C for 5 min, and then raised to 250°C at 25°C/min for 5 min. Mass conditions were as follows: electronic impact at 70 eV, interface temperature 280°C, emission current 200 µA, ion source temperature 230°C, scan range 40–450 m/z, and detector voltage 350 V. The qualitative identification of compounds was assigned by the retention indices (RI) and matching their recorded mass spectra with Wiley library and those stored in the NIST14 library of the GC–MS data system. The RI of the unknown compounds were determined via sample injection using a series of n‐alkanes (C6‐C30). To quantify the volatile compounds, peak areas were normalized with the internal standard 2‐octanol previously added to each sample. The relative volatile compound concentrations in samples were calculated by comparison with the concentration of the internal standard (2‐octanol). The concentration of the 4 kinds of bayberry wine volatile compounds was expressed as internal standard (2‐octanol) equivalents. Each sample was measured for four repeats.

### 2.5 Electronic nose analysis

The electronic nose (Germany Airsense PEN 3.5) was used to tentatively estimate the aroma profile similarity after fermentation. The E‐nose analysis was based on previous reports (Hong, Wang, & Qi, 2015; Li et al., 2017) and with modifications. The procedures were as follows: 5 ml of each bayberry wine sample was added in a 20‐ml glass vial and capped with a Teflon rubber cap. The vial with the bayberry wine sample was allowed to stand at room temperature for 30 min, while the headspace collected the volatiles from the wines. During the measurement process, the headspace gaseous compounds were pumped into the sensor arrays through a tube connected to a needle in the Teflon rubber cap at a flow rate of 400 ml/min, resulting in the ratio of conductance G/G0 (G and G0 are conductance of the sensors exposed to wine gas and zero gas, respectively) of each sensor changed.

The measurement time was 220 s, which was long enough for the sensors to reach stable signal values. When the measurement was completed, the data were stored by electronic nose software for later PCA and LDA analysis. After each samples, zero gas (air filtered by active carbon) was pumped into the sample gas path from the other port of the instrument for 120 s (flush time). The 10 metal oxide sensors of the PEN 3.5 electronic nose are described in Table 1. Different sensors respond to different volatile substances. Each wine sample was measured for five repeats.

**Table 1:** Chemical sensors used in electronic nose corresponding to different types of volatile substances

Sensor number	Sensor name	Sensor sensitives
1	W1C	Aromatic organic compounds
2	W5S	High sensitivity and sensitive with nitrogen oxides
3	W3C	Ammonia, a sensor for aromatic compounds
4	W6S	Mainly selective for hydrogen
5	W5C	Alkanes, aromatic compounds, and nonpolar organic compounds
6	W1S	Sensitive to methane. Broad range of organic compounds detected
7	W1W	Sensitive to sulfides, for example, H_2_S.
8	W2S	Detection of alcohol, partially sensitive to aromatic compounds, wide range
9	W2W	Aromatic compounds, sensitive to organic sulfides
10	W3S	Sensitive to alkanes, for example, high concentrations (>100 mg/kg) of methane and aliphatic organic compounds

### 2.6 Sensory evaluation of bayberry wine

Quantitative descriptive sensory analysis was applied for evaluation of the bayberry wine samples, using a scale from 0 to 9 (0 = none, 9 = highest intensity) (Niu et al., 2011; Stone, Sidel, Oliver, Woolsey, & Singleton, 1974). A panel of 11 trained assessors aged from 21 to 35 years, six males and five females, participated in sensory evaluation of the bayberry wines. The judges participated in the weekly sensory course (familiarization with evaluating aroma, flavor, and palate characteristics of the bayberry wines, and discussing and reaching consensus about the descriptive attributes). The sensory evaluation was conducted according to the reference standards (Sáenz‐Navajas, Campo, Fernández‐Zurbano, Valentin, & Ferreira, 2010), previous reports (Dias et al., 2017, Niu et al., 2011), and ISO 4,121. The sensory attributes consisted of fruity aroma, floral aroma, alcoholic aroma, sour, color, and overall acceptability. Bayberry wines were evaluated at controlled room temperature (20 –25°C) using ISO wine glasses. The sensory attribute data are presented as the mean of the scores provided by the 11 panel members. The sensory evaluation attribute reference standards of bayberry wine are shown in Table 2.

**Table 2:** List of sensory attributes and corresponding reference standards of bayberry wine

Attributes	Reference compositions
Color	20 ml bayberry juice
Fruity aroma	1 cm^2^ piece of fresh bayberry
	10 ml bayberry juice
	1 cm^2^ piece of fresh chopped pear, 10 ml pear juice
	1 cm^2^ piece of fresh chopped banana
Floral aroma	10 ml elderflower juice
Sour	Aqueous solution containing 0.07% citric acid
Alcoholic aroma	Aqueous solution containing 20% ethanol (food grade)
Overall acceptability	Balance word definition of the perceived balance between odor, taste, and mouthfeel

### 2.7 Statistical analysis

Data from the characterization of the bayberry wines are reported as mean ± standard deviation for quadruplicate determinations. Electronic nose measurements of bayberry wine sample were performed using WinMuster software (Winmuster1.6.2) for PCA and LDA. All the data were analyzed using the one‐way analysis of variance (ANOVA) using SPSS, version 22.0.

## 3 RESULTS AND DISCUSSION

### 3.1 Analysis of physicochemical properties of different bayberry wines

The content of the total sugar, soluble solids, anthocyanins, alcohol, titratable acidity, and pH of four bayberry wines was analyzed, and the data are listed in Table 3.

**Table 3:** Physicochemical properties of four bayberry wines

Physicochemical properties	DK wine	BQ wine	DA wine	WD wine
Total sugar (g/L)	6.42 ± 1.21^a^	6.31 ± 1.16^a^	6.13 ± 1.02^a^	6.46 ± 1.18^a^
Total soluble solids (°Brix)	7 ± 0.50^b^	7 ± 0.50^b^	7 ± 0.50^b^	8 ± 0.50^b^
Total anthocyanin content (mg/L)	51.1 ± 1.78^d^	88.5 ± 1.79^b^	72.8 ± 1.85^c^	116.6 ± 2.03^a^
Alcoholic strength (%vol.)	12.6 ± 0.49^a^	12.9 ± 0.52^a^	12.7 ± 0.58^a^	13.1 ± 0.65^a^
Titratable acidity (g/L)	9.30 ± 0.64^a^	6.63 ± 0.52^d^	8.56 ± 0.72^b^	8.18 ± 0.57^c^

Note

Different superscript letters in the same rows mean significant differences (*p* < .05).

It can be found that in the BQ wine, titratable acidity content was lowest, and the total sugar and alcohol content were moderate. Statistical analysis showed that there were significant differences (*p* < .05) in the total anthocyanin content between the bayberry wines made from the four cultivars. WD wine had the highest total anthocyanin content (116.6 mg/L) among the four kinds of bayberry wines. The other bayberry wines, in descending order by total anthocyanin content, were BQ wine (88.5 mg/L), DA wine (72.8 mg/L), and DK wine (51.1 mg/L). Anthocyanins are highly correlated to antioxidant capacity of the most fruits, which may have potential benefits for human health and disease prevention (Wallace, 2011; Wang et al., 2016). The present result showed that the variety of bayberry influenced the quality of bayberry wines.

### 3.2 Comparison of volatile flavor compounds of different bayberry wines

The volatile flavor compounds of the four kinds of bayberry wines are shown in Table 4. As can be seen from Table 4, a total of 46 volatile flavor compounds were found in the four kinds of bayberry wines, including 19 esters, 7 alcohols, 6 acids, 2 aldehydes, 2 ketones, 3 terpenes, and 7 others compounds. Most of the compounds were detected with high detection frequency in all four bayberry wines. Therefore, these compounds might play an important role in the characterization of bayberry wines.

**Table 4:** The concentration of volatile compounds identified in four kinds of bayberry wines

Code	Compounds	RI	Odor description	Concentration (mg/L)			
				DK wine	BQ wine	DA wine	WD wine
Esters							
A1	Ethyl acetate	605	Fruity, pineapple	1.258 ± 0.298^d^	3.462 ± 0.261^a^	2.287 ± 0.367^c^	2.863 ± 0.341^b^
A2	Ethyl butyrate	803	Fruity, pineapple	0.069 ± 0.018^b^	0.124 ± 0.032^a^	0.075 ± 0.015^b^	0.079 ± 0.009^b^
A3	Ethyl 2‐methylbutanoate	850	Fruity, apple	–	0.062 ± 0.005^a^	0.043 ± 0.002^b^	0.046 ± 0.004^b^
A4	Ethyl hexanoate	998	Fruity	0.249 ± 0.045^a^	0.261 ± 0.032^a^	0.251 ± 0.022^a^	0.263 ± 0.019^a^
A5	Ethyl pentanoate	898	Fruity, apple	0.032 ± 0.006^b^	0.049 ± 0.002^a^	–	0.042 ± 0.007^a^
A6	Ethyl heptanoate	1,097	Fruity, pineapple	0.501 ± 0.034^c^	0.826 ± 0.028^a^	0.647 ± 0.025^b^	0.692 ± 0.065^b^
A7	Ethyl octanoate	1,196	Fruity, banana, pear	2.054 ± 0.981^d^	6.245 ± 1.002^a^	4.069 ± 0.988^b^	3.912 ± 0.896^c^
A8	Ethyl nonanoate	1,294	Fruity, grape	0.306 ± 0.011^a^	0.282 ± 0.024^b^	0.214 ± 0.031^c^	0.341 ± 0.023^a^
A9	Ethyl phenylacetate	1,248	Rosy, honey	0.046 ± 0.012^b^	0.066 ± 0.003^a^	0.049 ± 0.007^b^	–
A10	Ethyl decanoate	1,394	Fruity	6.421 ± 1.993^c^	9.166 ± 2.046^a^	7.221 ± 1.331^b^	4.212 ± 1.024^d^
A11	Diethyl butanedioate	1,182	Fruity, apple	–	2.741 ± 0.186^a^	2.652 ± 0.235^a^	4.143 ± 0.211^b^
A12	Ethyl dodecanoate	1,493	Fruity	1.692 ± 0.341^c^	2.219 ± 0.532^a^	–	1.904 ± 0.565^b^
A13	Ethyl lactate	1,016	Fruity fatty	–	–	0.241 ± 0.006^a^	0.232 ± 0.011^a^
A14	Isoamyl acetate	878	Fruity, banana	0.082 ± 0.006^b^	0.096 ± 0.004^b^	0.162 ± 0.012^a^	0.256 ± 0.003^c^
A15	Diethyl succinate	1,176		0.041 ± 0.009^a^	–	0.045 ± 0.003^a^	0.047 ± 0.004^a^
A16	Benzoic acid, methyl ester	1,125	Fruity, floral aroma	0.033 ± 0.006^a^	0.027 ± 0.002^b^	–	0.024 ± 0.005^ab^
A17	3‐Nonenoic acid, methyl ester	1,223		–	–	–	0.041 ± 0.009
A18	Ethyl 3‐hydroxybutyrate	939	Alcoholic, solvent	–	0.061 ± 0.007^a^	0.057 ± 0.006^a^	0.047 ± 0.002^a^
A19	Formic acid, octyl ester	1,072	Fruity	–	0.026 ± 0.005^a^	–	0.019 ± 0.003^b^
	Total			18.407	25.713	18.013	19.163
Alcohols							
B1	2‐Methyl‐1‐butanol	1,203	Fruity	1.686 ± 0.098^c^	2.761 ± 0.076^a^	1.912 ± 0.56^b^	1.827 ± 0.64^b^
B2	Phenethyl alcohol	1,121	Rose fragrance	3.432 ± 0.87^d^	9.769 ± 1.23^a^	7.241 ± 1.53^c^	8.216 ± 1.76^b^
B3	1‐Hexanol	877	Fruity Floral aroma	–	0.057 ± 0.003^a^	0.054 ± 0.009^a^	0.060 ± 0.005^a^
B4	Terpinen‐4‐ol	1,187	Peppery earthy fragrance	0.426 ± 0.021^b^	–	0.524 ± 0.026^a^	0.462 ± 0.031^b^
B5	1‐Butanol	678	Alcoholic, solvent	0.043 ± 0.009^b^	0.057 ± 0.011^a^	0.054 ± 0.010^a^	0.048 ± 0.016^b^
B6	2‐Ethylhexanol	1,074	Sweet floral aroma	–	0.074 ± 0.012^a^	–	0.055 ± 0.021^b^
B7	Isoamyl alcohol	735	Fruity, apple	0.036 ± 0.007^c^	0.062 ± 0.015^a^	0.043 ± 0.010^b^	–
	Total			5.623	12.780	9.108	10.668
Acids							
C1	Acetic acid	1,453	Acidic	0.472 ± 0.023^a^	0.214 ± 0.018^d^	0.326 ± 0.022^b^	0.294 ± 0.031^c^
C2	Pentanoic acid	920	Sweaty, acidic	0.081 ± 0.014^a^	–	0.024 ± 0.009^b^	0.023 ± 0.005^b^
C3	2‐Methylbutanoic acid	876	Sweaty, acidic	0.041 ± 0.003^a^	0.029 ± 0.003^b^	0.032 ± 0.001^b^	0.039 ± 0.006^a^
C4	Hexanoic acid	1,022	Fatty, sweaty, acidic	0.052 ± 0.002	–	–	–
C5	Octanoic acid	1,288	Cheesy, fatty	0.189 ± 0.012^a^	0.066 ± 0.003^d^	0.076 ± 0.005^c^	0.098 ± 0.015^b^
C6	Decanoic acid	1,370	Fatty, sweaty, acidic	0.072 ± 0.006^a^	0.014 ± 0.003^b^	–	0.016 ± 0.001^b^
	Total			0.907	0.323	0.458	0.470
Aldehydes							
D1	Phenylacetaldehyde	1,055	Fruity, sweet	–	0.053 ± 0.007^a^	0.041 ± 0.011^b^	0.039 ± 0.004^b^
D2	Benzaldehyde	982	Almond	0.026 ± 0.003^c^	–	0.028 ± 0.009^b^	0.075 ± 0.005^b^
	Total			0.026	0.053	0.069	0.114
Ketones							
E1	3‐Hydroxy‐2‐butanone	721	Sweet	0.021 ± 0.001^a^	0.025 ± 0.006^a^	0.026 ± 0.004^a^	0.020 ± 0.002^a^
E2	6‐Methyl‐5‐hepten‐2‐one	995	Fruity	0.133 ± 0.006	–	–	–
	Total			0.154	0.025	0.026	0.020
Terpenes							
F1	Caryophyllene	1,430	Woody citrus aroma	1.021 ± 0.17^d^	2.631 ± 0.28^a^	1.256 ± 0.23^c^	1.543 ± 0.31^b^
F2	γ‐Terpinene	1,067	Fruity	–	0.042 ± 0.004^b^	0.052 ± 0.009^a^	0.043 ± 0.006^b^
F3	2‐Carene	1,095		0.045 ± 0.006^a^	0.027 ± 0.002^b^	0.042 ± 0.005^ab^	–
	Total			1.066	2.700	1.350	1.586
Others							
G1	5‐Ethyl‐2(5H)‐furanone	956		0.089 ± 0.008^a^	0.072 ± 0.007^b^	0.069 ± 0.004^b^	0.071 ± 0.003^b^
G2	2‐Pentyl‐furan	1,014		0.056 ± 0.009^a^	0.055 ± 0.006^a^	0.049 ± 0.006^a^	0.052 ± 0.050^a^
G3	3,3,5‐Trimethyl‐1,5‐heptadiene	1,057		0.044 ± 0.005^a^	–	0.044 ± 0.006^a^	0.047 ± 0.002^a^
G4	Butylcyclohexane	1,037		–	0.080 ± 0.009^a^	0.057 ± 0.005^b^	0.032 ± 0.003^c^
G5	Methylcyclohexane	842		0.180 ± 0.006^a^	0.080 ± 0.004^b^	0.075 ± 0.008^b^	0.079 ± 0.006^b^
G6	3‐Heptene	1,099		–	0.429 ± 0.003^a^	0.233 ± 0.007^b^	0.156 ± 0.005^c^
G7	o‐Cymene	1,028		0.425 ± 0.09^c^	0.529 ± 0.12^a^	0.432 ± 0.06^c^	0.469 ± 0.07^b^
	Total			0.794	1.245	0.959	0.906

Note

The symbol "–" represents nondetected; different superscript letters in the same rows mean significant differences (*p* < .05); Aroma Description Reference Website http://www.thegoodscentscompany.com/.

**Figure 1:**
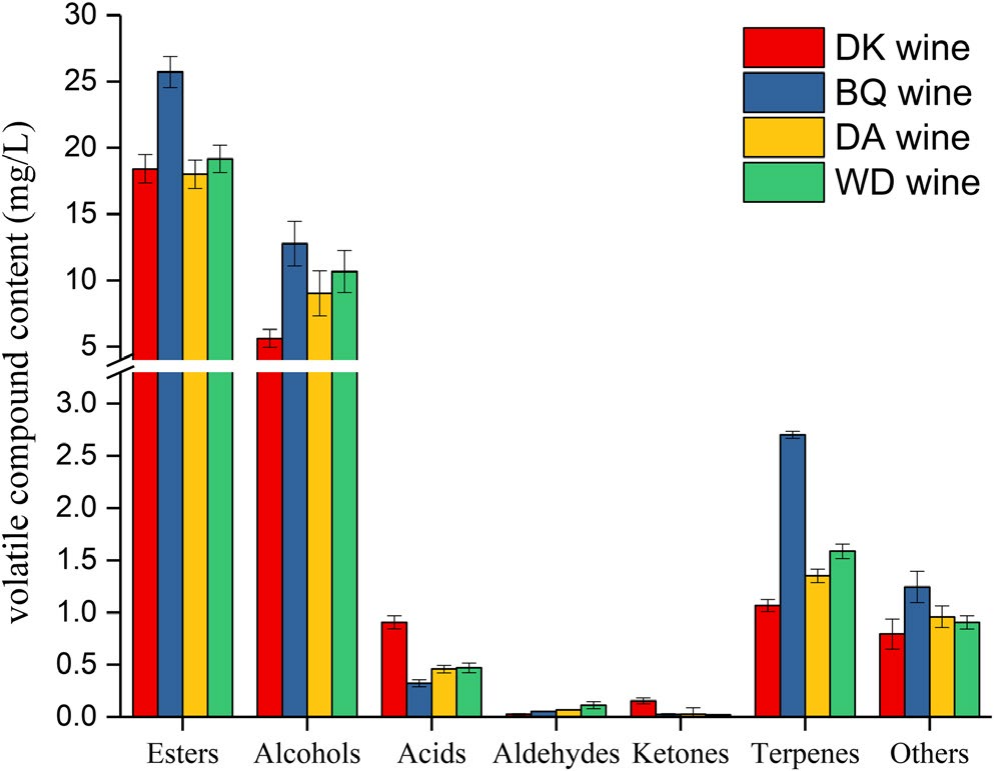
The concentration of volatile compounds identified in 4 different types of bayberry wines

During fermentation, yeasts convert sugar to ethanol, producing a variety of by‐products such as higher esters, alcohols, acids, aldehydes, ketones, terpenes, and other volatile compounds which contribute to wine aroma (Styger, Prior, & Bauer, 2011). As shown in Figure 1, esters and alcohols were the largest groups, the main aroma compounds in bayberry wines. They were produced during alcoholic fermentation and played an essential role in wine flavor, depending on types of compounds and their concentrations (Valero et al., 2002).

Esters of four bayberry wines were mainly composed of ethyl acetate, ethyl hexanoate, ethyl heptanoate, ethyl octanoate, ethyl nonanoate, ethyl decanoate, diethyl butanedioate, ethyl dodecanoate, and isoamyl acetate. Esters can impart bayberry wines fruity and floral aromas (Liu et al., 2019). It has been suggested that esters are formed mainly through the esterification of alcohols with organic acids during the fermentation and storage processes (Erten, Tanguler, & Cakiroz, 2007). The content of ester compounds in the BQ wine was the highest (total 25.713 mg/L). The main esters in BQ wine are ethyl decanoate (9.166 mg/L), ethyl octanoate (6.245 mg/L), ethyl acetate (3.462 mg/L), diethyl butanedioate (2.741 mg/L), and ethyl dodecanoate (2.219 mg/L), respectively. Ethyl hexanoate has a fruity and wine‐like aroma. Ethyl octanoate has a fruity and banana aroma, and ethyl acetate and ethyl decanoate have a fresh fruity aroma (Ayestarán et al., 2019). Ethyl pentanoate and ethyl heptanoate are associated with fruity and apple notes (Fan & Qian, 2006).

Alcohols were the second abundant group of volatile compounds in the different varieties of bayberry wine. Most of the alcohols found in bayberry wines are higher alcohols. A total of seven higher alcohols were detected as the major flavor compounds in the fermented bayberry wine, which is 2‐methyl‐1‐butanol, phenethyl alcohol, 1‐hexanol, terpinen‐4‐ol, 1‐butanol, 2‐ethylhexanol, and isoamyl alcohol. The highest content of higher alcohols was BQ wine (12.780 mg/L), followed by WD wine (10.668 mg/L), DA wine (9.108 mg/L), and DK wine (5.623 mg/L). The content of higher alcohols in the four kinds of bayberry wine was dominated by phenylethyl alcohol, and the highest content of phenylethyl alcohol in BQ wine was 9.769 mg/L. Alcohols impart a special aroma to the wine, such as phenylethyl alcohol with a light rose aroma and 2‐methyl‐1‐butanol with a light fruity aroma (Francis & Newton 2008). The alcohols were mainly formed in the fermentation process and played a significant role in the bayberry wine aroma profile.

In this study, the major volatile acids (acetic acid, pentanoic acid, 2‐methylbutanoic acid, hexanoic acid, octanoic acid, and decanoic acid) were identified in the four kinds of bayberry wines. The total of acids are 0.907 mg/L (DK wine), 0.323 mg/L (BQ wine), 0.458 mg/L (DA wine) and 0.470 mg/L (WD wine), respectively. The results showed that the acetic acid was the most important volatile acid of the total acids. The glucose was transformed to acetic acid and ethanol by yeast metabolism during fermentation (Pinto, Malfeito‐Ferreira, Quintieri, Silva, & Baruzzi, 2019). In general, low levels of volatile acids are ideal for producing high‐quality wines. Acetic acid content is an important factor affecting the quality of fermented wine. Excessive 0.7 g/L will produce pungent odor and bad taste (Mains 2014).

Aldehydes and ketones were another key aroma group in bayberry wine. Phenylacetaldehyde, 3‐hydroxy‐2‐butanone, and 6‐methyl‐5‐hepten‐2‐one have sweet and fruity aroma. Benzaldehyde possesses an almond aroma. Terpenes have great benefits for the human body (Petrović, Stojković, & Soković, 2019). Biqi bayberry wine contained more amounts of terpenes (total 2.700 mg/L) than DK wine, DA wine, and WD wine. Caryophyllene has a sweet woody and with a citrus background aroma.

### 3.3 Electronic nose analysis

**Figure 2:**
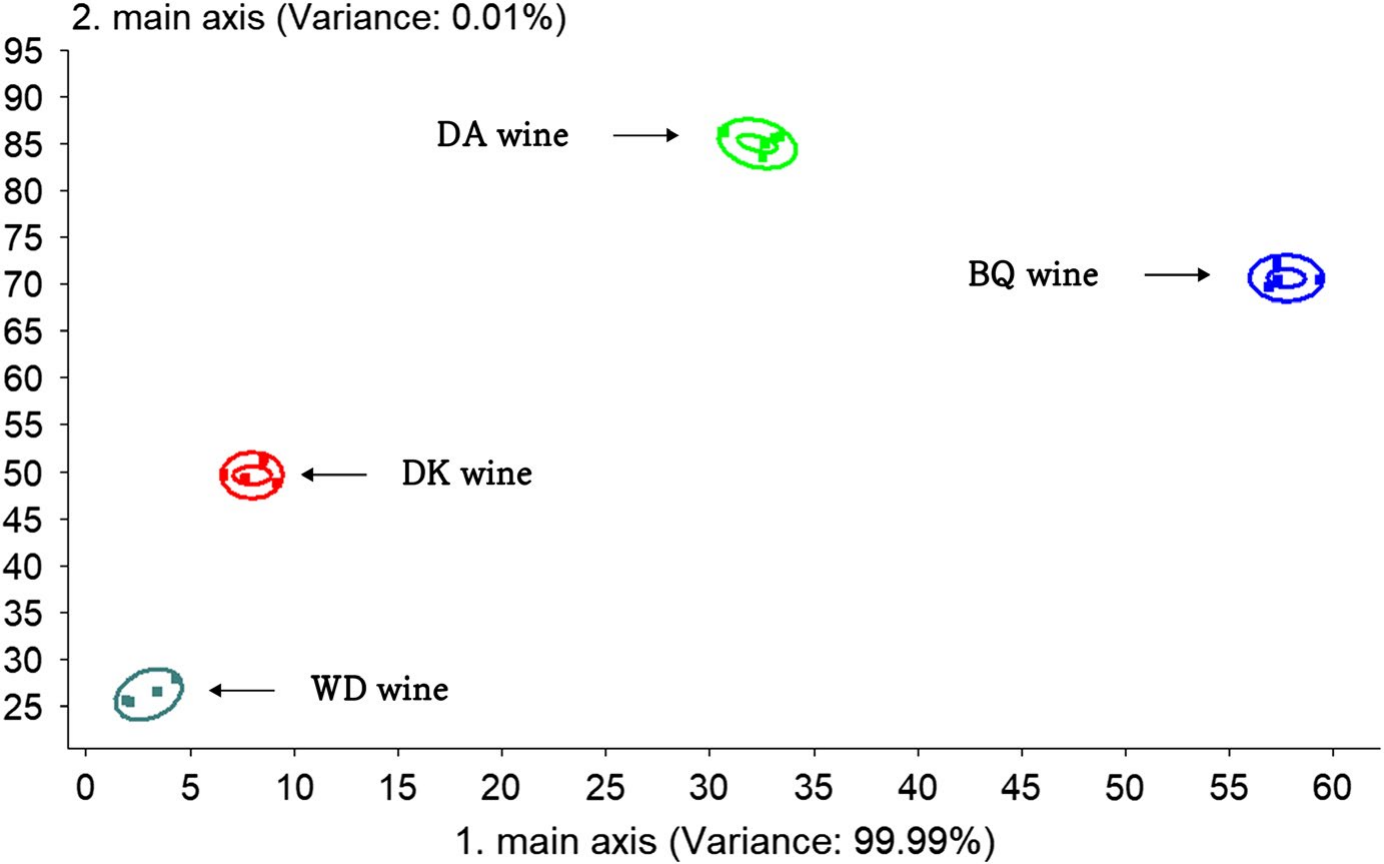
PCA of four kinds of bayberry wines

**Figure 3:**
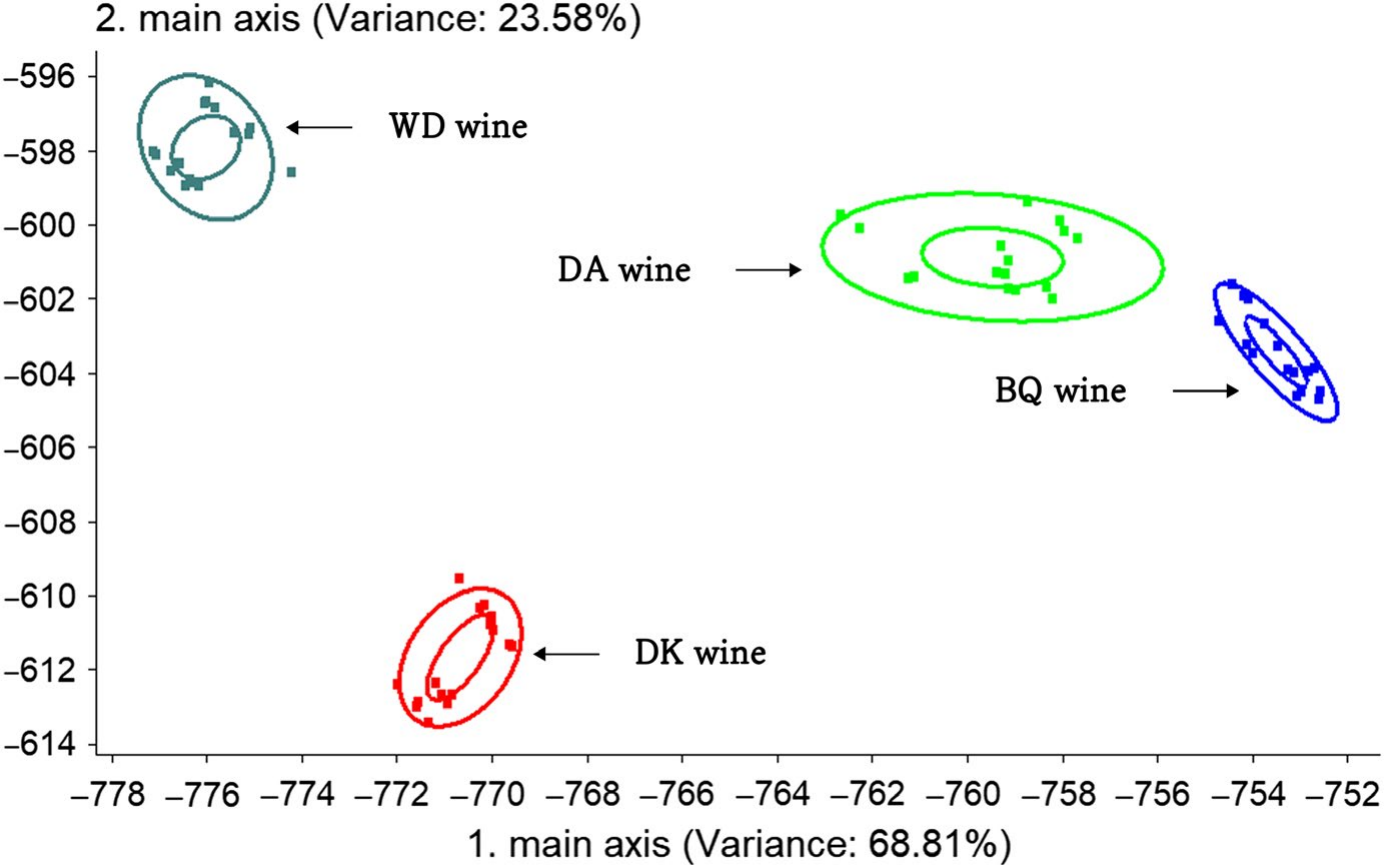
LDA of four kinds of bayberry wines

Principal component analysis (PCA) is a method for studying the similarities and differences between various measurement data (Huang, Wu, Chen, Weng, & Zhang, 2018). The bayberry wine samples were separated along the first principal component (PC), which described 99.99% of the variance contribution rate (Figure 2, PCA), and showed four defined groups. Along the PC1 axis, the BQ wine group was located with high positive scores. The total variance contribution rate indicating information through PCA analysis could reflect the difference of four kinds of bayberry wine. Linear discriminant analysis (LDA) studies the distribution of different samples and their distances to each other in order to distinguish different samples (Sun et al., 2018). The total contribution rate of the two linear discriminant functions (LD) reached 92.39%. A data point plot (Figure 3, LDA) was depicted with LD1 and LD2, whose variance contribution rate was of 68.81% and 23.58%, respectively. The center distance of the four kinds of bayberry wine group is far from each other. Owing to the high variance contribution rate, sufficient representativeness can be observed (Li et al., 2017). The results show that the volatile flavor compounds of the four kinds of bayberry wines had obvious differences. This is consistent with the results detected by GC‐MS.

### 3.4 Sensory evaluation

**Figure 4:**
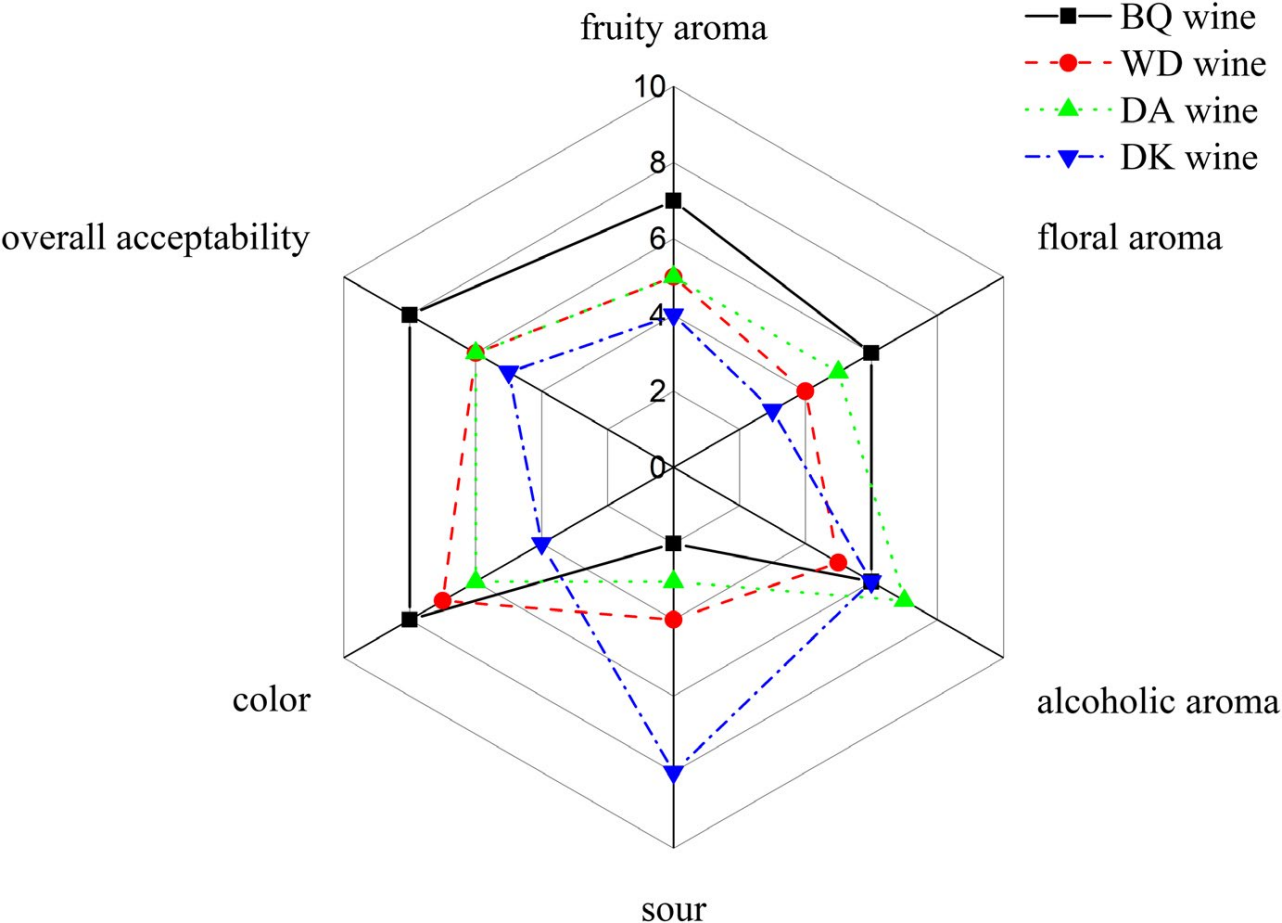
Graph of the mean sensory scores of the four bayberry wines studied

The results of the sensory evaluation analysis are shown in Figure 4.

The result analysis demonstrated that “fruity aroma,” “floral aroma,” “alcoholic aroma,” “sour,” “color,” and “overall acceptability” descriptors showed significant differences between the four kinds of bayberry wines in the sensory evaluation scores. Dongkui bayberry wine was intense in “sour” descriptors, whereas “floral aroma” and “color” exhibited lower level. Dongkui bayberry wine tasted the sourest due to a lot of acetic acid. Acetic acid played an important role in the formation of fruit wine flavor substances, but excessive acetic acid causes the wine too acidic when tasted. In addition to alcoholic aroma, BQ wine exhibited obviously fruity aroma and floral aroma, and it may be related to the high relative content of esters and higher alcohols. Moreover, BQ wine has beautiful color and good overall acceptability. From the sensory evaluation, the sensory quality of the BQ wine was the best among the four bayberry wines.

## 4 CONCLUSIONS

The analysis of sensory, physicochemical properties and volatile flavor compounds of the different bayberry wines by GC‐MS coupled with E‐nose have shown that the variety of bayberry has a greater effect on the quality of bayberry wines under the same brewing process. Esters and alcohols were the main aroma compounds in bayberry wines. Moreover, the different bayberry wines aroma feature could well be distinguished based on GC–MS results and PCA and LDA of E‐nose data. Based on the comprehensive results, it is advisable to select the BQ in the eastern Zhejiang province for the fermentation of the bayberry wine. The fact of this study may provide an important basis for selection of brewing materials of high‐quality bayberry wines.

## CONFLICT OF INTEREST

The authors have no conflicts of interest.

## ETHICAL APPROVAL

This study does not involve any human or animal testing.
